# Convention of Medical Teachers

**Published:** 1860-08

**Authors:** 


### Convention of Medical Teachers.

The effort upon the part of the *tricksters* engaged in medical teaching and those desiring such a position, to induce the Medical Colleges of the country to assemble in Convention and submit to the *dictation* of the so-called American Medical Association, has again proved a most signal failure—*not one* of the most prominent Medical Colleges in the country being represented. As the representative of the Atlanta Medical College, we went to New Haven to have a *conference* with those engaged most prominently in medical teaching. Finding a different body from that to which we were accredited, we offered a preamble and resolutions, which, in our judgment, stated the facts and met the circumstances, which did not, however, meet the approbation of these representatives of the *smaller* schools of the country, who have undertaken to initiate a plan by which every Medical College in this broad land, from Maine to California, shall be virtually *compelled* to conform to a uniform system of medical education in every particular. Knowing our own rights, and being determined to pursue the course dictated by our own sense of right and propriety, with a disposition, at the same time, to compromise, and make an honest effort for the adoption of means holding out a reasonable prospect for the elevation of the standard of medical education, we were not disposed to assume the right to initiate a plan of medical education intended to operate through the American Medical Association upon all the colleges of the country, without the direct and active concurrence of those institutions, which from their age, position and patronage, it would be presumed had some control and influence over the subject. Holding these views, and regarding the claim, upon the part of the Association, to regulate this matter, as the greatest possible absurdity, we formally withdrew from what we regarded as an unauthorized and impotent fragment of the colleges of the country, acting to a large extent under influences antagonistic to the Institution with which we are connected.

